# Revisiting the Prognostic Significance of CD155 in Invasive Breast Carcinoma

**DOI:** 10.22034/ijp.2026.2087963.3664

**Published:** 2026-08-01

**Authors:** Burcu Sanal Yılmaz

**Affiliations:** 1 Department of Pathology, Faculty of Medicine, Karamanoğlu Mehmetbey University, Karaman, Türkiye

## Dear Editor,

We read with great interest the article by Abdelkerim et al., entitled “CD155 Expression in Invasive Breast Carcinoma: Association with Immune Tumor Microenvironment,” recently published in the Iranian Journal of Pathology. The authors should be commended for evaluating the relationship between CD155 expression, CD56-positive tumor-infiltrating natural killer (NK) cells, CD163-positive tumor-associated macrophages, and clinicopathological parameters in invasive breast carcinoma. Their findings contribute valuable information regarding the potential role of CD155 within the breast cancer immune microenvironment and provide a basis for further investigation of its biological and clinical significance (1).

One aspect that may warrant further consideration concerns the interpretation of the prognostic significance of CD155 expression. Although CD155 positivity was significantly associated with several adverse clinicopathological features, including larger tumor size, higher histological grade, lymphovascular invasion, nodal metastasis, advanced stage, and unfavorable molecular subtypes (1), the study did not include follow-up data or survival analyses. Therefore, while the findings strongly suggest an association with aggressive tumor characteristics, the conclusion that CD155 may serve as a poor prognostic indicator should perhaps be interpreted with some caution. Previous studies have reported prognostic implications of CD155 expression in breast cancer and other malignancies (2, 3); however, additional investigations incorporating disease-free and overall survival analyses would be valuable to determine whether CD155 represents an independent prognostic biomarker or primarily reflects aggressive tumor biology.

Another particularly interesting observation was the association between increased densities of CD56-positive tumor-infiltrating NK cells and adverse clinicopathological characteristics (1). Although NK cells are traditionally regarded as important mediators of antitumor immunity, accumulating evidence suggests that persistent exposure to the tumor microenvironment may lead to functional impairment and exhaustion of NK-cell populations. Because CD56 primarily reflects cellular presence rather than functional activity, quantitative assessment alone may not fully characterize the biological significance of NK-cell infiltration. Evaluation of functional markers such as NKG2D, TIGIT, granzyme B, or perforin may provide additional insight into whether increased NK-cell density reflects an effective immune response or the accumulation of functionally compromised NK cells (4).

Recent evidence further highlights the biological complexity of the CD155 axis in breast cancer. Ou et al. demonstrated that the CD155/CD226/CD96/TIGIT immune checkpoint network provides substantial prognostic information and may serve as a clinically relevant predictor of postoperative survival, emphasizing that CD155 functions within a broader immunoregulatory context rather than as an isolated biomarker (5). Furthermore, Zhao et al. reported that CD155 expression in triple-negative breast cancer was positively associated with lymph node metastasis, Ki-67 proliferation index, and CD163-positive macrophage infiltration, supporting its close relationship with an immunosuppressive tumor microenvironment and adverse clinical outcomes. These observations reinforce the view that the prognostic significance of CD155 should be interpreted within the context of its interactions with immune checkpoint pathways and tumor-associated immune cell populations rather than on the basis of CD155 expression alone (6).

In conclusion, we congratulate the authors for their valuable contribution to the understanding of CD155 expression and immune-cell interactions in invasive breast carcinoma. Additional studies incorporating survival analyses, functional characterization of immune-cell subsets, and comprehensive evaluation of the CD155–TIGIT/CD96/CD226 immune checkpoint axis are warranted before the prognostic significance of CD155 can be fully established (5, 6, 7). Such investigations may further clarify the biological and therapeutic relevance of this pathway in breast cancer.

## Data Availability

Data sharing is not applicable to this article.
